# Family Medicine Training in Lesotho: A Strategy of Decentralized Training for Rural Physician Workforce Development

**DOI:** 10.3389/fmed.2020.582130

**Published:** 2021-01-14

**Authors:** Benjamin Bryden, Mariel Bryden, Jonathan Steer-Massaro, Sebaka Malope

**Affiliations:** ^1^Lesotho Boston Health Alliance Family Medicine Specialty Training Program, Leribe, Lesotho; ^2^Ministry of Health Lesotho, Maseru, Lesotho; ^3^Department of Family Medicine, Boston University School of Medicine, Boston, MA, United States; ^4^Department of Obstetrics and Gynecology, Boston University School of Medicine, Boston, MA, United States

**Keywords:** Lesotho, family medicine, primary care education, family medicine training, rural training

## Abstract

Family medicine is a relatively new but rapidly expanding medical discipline in Sub-Saharan Africa. Specialization in family medicine is an effective means for building and retaining a highly skilled rural physician workforce in low- and middle-income countries. The Lesotho Boston Health Alliance Family Medicine Specialty Training Program is the first and only postgraduate family medicine program and the only accredited postgraduate training program in the Kingdom of Lesotho. Lesotho has unique challenges as a small mountainous enclave of South Africa with one of the lowest physician-to-patient ratios in the world. Most health professionals are based in the capital city, and the kingdom faces challenging health problems such as high human immunodeficiency virus prevalence, high maternal mortality, and malnutrition, as well as increasing burdens of non-communicable diseases such as hypertension, diabetes, and obesity. In response to these health crises and the severe shortage of health professionals, Lesotho Boston Health Alliance partnered with the Lesotho Ministry of Health in 2008 to introduce family medicine as a new specialty in order to recruit home and retain Basotho doctors. Family medicine training in Lesotho uses a unique decentralized, non-university-based model with trainees posted at rural district hospitals throughout the country. While family medicine in Lesotho is still in the early stages of development, this model of decentralized training demonstrates an effective strategy to develop the rural health workforce in Lesotho, has the potential to change the physician workforce and health care system of Lesotho, and can be a model for physician training in similar environments.

## Background

Specialization in family medicine (FM) is an effective means of building and retaining a highly skilled rural physician workforce in low- and middle-income countries. FM is a relatively new but rapidly expanding medical discipline in Sub-Saharan Africa, and several postgraduate training programs in FM have started in the last several years or are in the process of planning for enrollment (1–3).

The Lesotho Boston Health Alliance (LeBoHA) Family Medicine Specialty Training Program (FMSTP) is the first and only accredited postgraduate medical education program in the Kingdom of Lesotho and was started in 2008. Lesotho is a predominantly rural country with mountainous terrain, making travel and health care delivery challenging. Thus, it offers an ideal setting for decentralization of health care services and training of a highly skilled rural physician workforce. In Lesotho, rural is broadly defined as outside of the capital city of Maseru, which is the main urban center of the Kingdom. Lesotho has nine predominantly rural districts, each with a small capital and a district hospital that oversees several rural health centers and health posts (4).

## Context

Lesotho is a small enclave country within South Africa and as a nation faces major health systems challenges including a predominantly centralized health services model. Although the population is primarily rural, the majority of the country's health professionals, health care budget, and single tertiary hospital are based in the capital city of Maseru. (5). The nation has the world's second highest human immunodeficiency virus prevalence, affecting ~25% of adults, high maternal and infant mortality, widespread malnutrition, and increasing burdens of non-communicable diseases such as diabetes, hypertension, and obesity (4, 6). Lesotho ranks 164 out of 189 on the United Nations Human Development Index (7). In 2017, Lesotho had a ratio of doctors to population of ~0.9 per 10,000, and a nurse-midwife to population ratio of 10.2 per 10,000, about one-third of the African average for doctor ratio to population and well below the World Health Organization–desired doctor–population ratio of 1:1,000 (8, 9). With no medical school in Lesotho, Basotho students must leave the country to train in South Africa and other countries, where many choose to remain after their education because of higher salaries, better working conditions, and more comfortable living conditions (10). The poor retention of Basotho doctors is complex; difficult working conditions, little opportunity for continuing education, isolation in rural hospitals, limited opportunities for advancement or career development, and challenges from working in a poorly functioning health care system are all major contributing factors. Because of these challenges and the enormous burden physicians face, unsurprisingly burnout and poor attrition are common (11).

## Project Description

LeBoHA was created as a partnership between Boston University and the Lesotho Ministry of Health (MOH) in response to this health crisis and the severe shortage of health professionals, particularly where they are needed most in rural areas. Boston University has partnered with the Lesotho MOH since the 1990s to support the Lesotho health care system through capacity building, human resource development, and strengthening of civil society. This long-term partnership culminated in the formation of LeBoHA in 2001 and the development of the FMSTP in 2008. The purpose of the FSMTP is to respond to the severe shortage of health care professionals in Lesotho by developing the human resources to improve and sustain high quality and comprehensive health care throughout the country. The FMSTP is a 4 year, part-time FM training program that allows for specialization in FM. The program is multidimensional and involves partnerships with the Lesotho government, including the Ministries of Health and Education, educational accrediting bodies including the Lesotho Council of Higher Education, multiple non-governmental organizations, and several regional universities. Building on the success of the FMSTP, in 2019 the Lesotho MOH, in partnership with LeBoHA, initiated a medical internship program furthering its effort to develop and retain a local physician workforce.

FM training in Lesotho has been adapted to the local environment and includes comprehensive, broad-spectrum training in clinical medicine, community-oriented primary care, district health management, operational health systems research, public health, quality improvement, leadership, and clinical governance. The FMSTP uses a teaching model that involves monthly didactic training at weeklong contact sessions for first- and second-year registrars (doctors enrolled in this program), focused primarily on clinical medicine topics, and quarterly weeklong contact session for third- and fourth-year registrars, focused on public health, leadership and management, and research skills. Contact sessions are held at Motebang Hospital, the largest district hospital in the country. Registrars complete supervised clinical rotations alongside local physicians as well as visiting regional and international specialists. Family physicians in Lesotho are upskilled in certain critical areas such as obstetrics and gynecology, diagnostic imaging including ultrasound, and surgical skills. Supervised clinical rotations are geared toward providing registrars opportunities to learn specialized skills and procedures previously only available at the referral level in order to decentralize some of these to the district level. Additionally, they complete a community-oriented primary care project, a quality improvement initiative, and a research thesis. As registrars are based throughout the country, this network of FM trainees allows best practices to be quickly disseminated throughout the country.

The majority of learning occurs at the facility where a registrar works and is guided by a portfolio of learning and quarterly site supervision by faculty consultants. These supervision visits of the registrars in their home facilities focus on both their clinical and non-clinical activities, with special attention paid to how registrars are incorporating their new knowledge and skills at their facilities. The portfolio of learning serves as a record of a registrar's progress through the program and a roadmap of what they still have to learn, enabling self-directed learning with support from program faculty as well as local mentors. The vast majority of time in training is spent at the district level in keeping with the program's goal to train doctors to work in a rural environment.

The program enrolls physicians of all nationalities who are practicing in Lesotho who have completed internship, have a commitment to staying long term in Lesotho, and are employed in all sectors (government, Christian Health Association of Lesotho, and private). Basotho physicians employed by the government are preferentially accepted into the program, and the government of Lesotho sponsors the tuition of Basotho nationals. All current graduates of the program are Basotho nationals, with the majority retained as government civil servants. The majority of those enrolled in the program currently are Basotho nationals and government civil servants. Many registrars hold leadership positions as medical superintendent or district medical officer.

## Discussion

The introduction of FM as a new specialty is a strategy to not only retain and recruit doctors to live and work in Lesotho, but specifically to ensure there are highly skilled doctors in rural areas where high-quality health care is most needed but currently least available. In Lesotho, similar to other countries in the region, most doctors are generalist medical officers who have completed medical school and 1 to 2 years of internship prior to practicing. The LeBoHA FMSTP is unique in that family physicians are trained in a decentralized environment outside of a national university, although the program is a fully accredited postgraduate academic institution. Registrars in the program are trained comprehensively in primary health care to act as leaders of their primary health care team and are also trained as consultants to be an expert in services and procedures that are available at the district level.

FM is an officially recognized medical specialty in Lesotho, and after graduation, doctors in the government system are eligible for promotion to the consultant grade, the highest level of pay for civil service in Lesotho. The LeBoHA FMSTP provides opportunities for continued career development for graduates, and these benefits are proving effective in motivating the training and retention of highly skilled rural family physicians. Although the program is relatively young, all graduates thus far have been retained in Lesotho, with the majority remaining in rural areas and in government service. The program has nine graduates including three who completed in 2020. A qualitative study on job satisfaction of family graduates of the program is planned for the future (Table 1).

**Table 1:** Profile of FMSTP graduates.

	**Graduation date**	**Sex**	**Nationality**	**Current position**	**Employer**	**Location**
Graduate 1	2011	M	Mosotho	FMSTP Director	Lesotho MOH	Leribe District
Graduate 2	2013	F	Mosotho	MOH Director of Clinical Services	Lesotho MOH	Maseru District
Graduate 3	2019	M	Mosotho	Private Practice, Family Physician	Private	Mafeteng District
Graduate 4	2019	F	Mosotho	District Medical Officer	Lesotho MOH	Butha Buthe District
Graduate 5	2019	F	Mosotho	District Medical Officer	Lesotho MOH	Mokhotlong District
Graduate 6	2019	F	Mosotho	Internship Deputy Director	Lesotho MOH	Maseru/Leribe District
Graduate 7	2020	M	Mosotho	Medical Officer/Family Physician	Lesotho MOH	Qacha's Nek District
Graduate 8	2020	F	Mosotho	Family Physician	NGO	Maseru District
Graduate 9	2020	M	Mosotho	District Medical Officer	Lesotho MOH	Leribe District

While medical schools in Sub-Saharan Africa and around the world are starting to decentralize clinical training at the undergraduate and internship level due to strains on tertiary institutions, it is unusual for postgraduate training to occur at the community level in a rural environment (12–14). Although FM is a dynamic field where the scope of skills and training of family doctors varies depending on context, throughout the world family physicians are employed in rural environments, and there are models for community training of family doctors in settings such as Brazil, Cuba, Australia, and North America in a community setting (15). While other postgraduate FM training programs in the region primarily offer university-based training, the program in Lesotho challenges this model by retaining postgraduate trainees in the environment where they are intended to work and be leaders. Additionally, the FMSTP demonstrates that postgraduate medical education can occur without a local medical school. There are challenges with this model, such as difficulties in cultivating an academic environment and lack of multidisciplinary support. Despite these challenges, we believe that training family physicians in the environment where they are most needed is the best way to retain physicians and improve the health care system from within.

## Conclusions

FM as a field shifts the focus of health care from the disease to the person, and from the hospital to the community. Decentralizing the training of family physicians out of the tertiary and university settings and into districts and often isolated communities offers a model of how to make these shifts. The LeBoHA FMSTP demonstrates this model in action and thus far has been successful in retaining physicians within the country and largely in the public sector. As more family physicians graduate and work in rural environments in Lesotho, the program's impact will need to be evaluated, and future research will need to be done on retention of family physicians in Lesotho and in rural areas and the impact of family physicians on the health care system.

## Data Availability Statement

The original contributions presented in the study are included in the article/supplementary material, further inquiries can be directed to the corresponding author/s.

## Author Contributions

BB is faculty for the program described in the article and wrote the initial draft of the manuscript. MB and JS-M are faculty for the program described in the article and wrote and edited key sections of the manuscript. SM is the program director of the program described in the article and wrote and edited key sections of the manuscript. All authors contributed to the article and approved the submitted version.

## Conflict of Interest

The authors declare that the research was conducted in the absence of any commercial or financial relationships that could be construed as a potential conflict of interest.
